# Loose ends in the differential diagnosis of IBS-like symptoms

**DOI:** 10.3389/fmed.2023.1141035

**Published:** 2023-07-06

**Authors:** Greger Lindberg, Ghazaleh Mohammadian

**Affiliations:** ^1^Department of Medicine at Huddinge, Karolinska Institutet, Stockholm, Sweden; ^2^Neurogastroenterology Unit, Division of Gastroenterology, Department of Gastroenterology, Dermatovenereology and Rheumatology, Karolinska University Hospital, Stockholm, Sweden

**Keywords:** irritable bowel syndrome, hypermobility spectrum disorders, autism spectrum disorders, diagnosis, symptom criteria

## Abstract

Two thirds of the patients we believed to have IBS in the 1970’s have since been possible to diagnose with treatable conditions like bile acid diarrhea, inflammatory bowel disease, microscopic colitis, celiac disease, disaccharide malabsorption, exocrine pancreatic insufficiency, or rare genetic variants. Despite advances in diagnostic techniques a substantial proportion of patients continue suffering from IBS-like symptoms that cannot be explained by current knowledge. Although it is likely that further research will reveal small but important subgroups of patients with treatable mechanisms for IBS-like symptoms, we propose that only two large groups remain for being addressed in the clinic: those with connective tissue disorders such as Ehlers-Danlos syndrome or hypermobility spectrum disorders and those with autism spectrum disorders. Patients with connective tissue disorders exhibit identifiable disturbances of gut motor function and possibly increased gut permeability as underlying mechanisms for IBS-like symptoms. Autism spectrum disorders pose a much more difficult problem in the clinic. Disturbances of perception combined with anxiety and excessive worry about signals from the gut can lead to an endless but futile search for something being wrong. The search can involve large numbers of care givers, no one understanding the patient’s suffering. Others may try to change their diet to lessen symptoms, only to find that almost all foods may cause worrying perceptions from the gut. Early recognition of autism spectrum disorders is essential for finding better ways to help patients with gastrointestinal and, as is often the case, extraintestinal symptoms.

## 1. Introduction

The irritable bowel syndrome (IBS) has been used as a diagnostic label for patients with abdominal pain and disturbances of bowel function but no detectable disease explaining the symptoms for more than 70 years (1). The gastrointestinal tract has a limited language and symptoms of IBS will inevitably overlap with those from many treatable organic conditions. In 1978 a group of British researchers compared symptoms between 32 patients with IBS and 33 patients with various organic gastrointestinal diseases (2). They found that four symptoms were significantly more common among patients with IBS: looser stools at onset of pain; more frequent bowel movements at onset of pain; pain often eased after bowel movement; and visible distension. Mucus per rectum and a sensation of incomplete evacuation were also more common in these patients. The key feature of Manning’s study was that it took patients with clinically defined IBS as the starting point. This approach was also taken by Kruis et al. (3), who developed a more elaborate diagnostic score based on logistic regression of data from 108 patients with clinically defined IBS and 209 patients with organic diseases. However, the main problem with the above and similar works was that there was no agreement on a universal definition of IBS.

**Figure 1:**
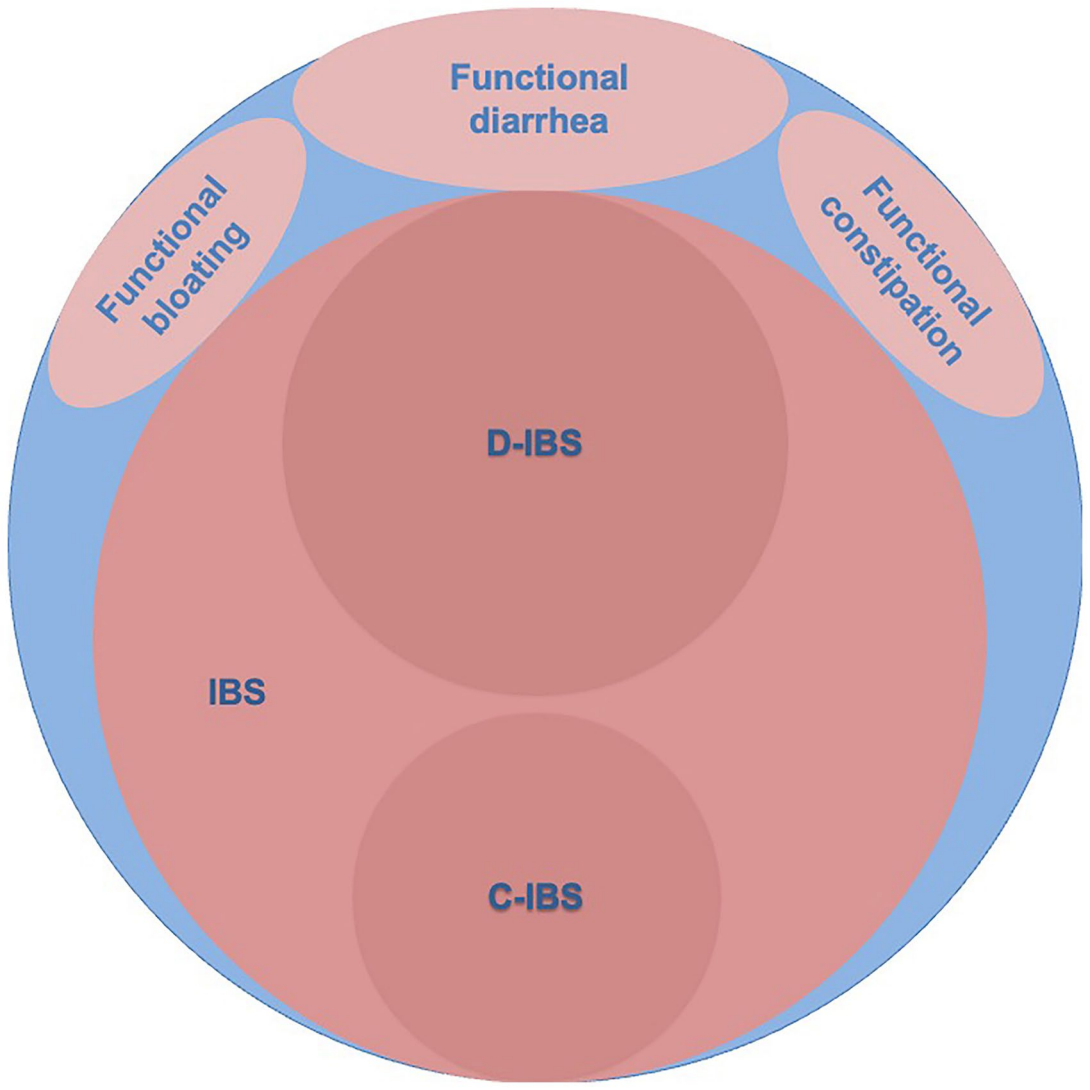
The blue area represents clinically recognized irritable bowel syndrome (IBS). The light red areas represent diagnoses according to Rome-II criteria and the dark red areas indicate subgroups of diarrhea-predominant (D-IBS) and constipation-predominant (C-IBS) irritable bowel syndrome according to Rome-II criteria (5).

Later work took a different view by defining criteria that, if satisfied, implied that the patient might have IBS [Rome Criteria (4), Rome-II criteria (5), Rome-III (6), and recently Rome-IV (7)]. By doing so, the new definitions of IBS came to exclude some of the patients with clinically recognized IBS, but criteria were purported to identify more homogeneous subgroups. Patients that did not fit the new definitions received other diagnostic labels like Functional abdominal bloating, Functional diarrhea, Functional constipation, or Unspecified functional bowel disorder (Figure 1). IBS was further subclassified according to the stool pattern into diarrhea-predominant IBS (D-IBS), constipation-predominant IBS (C-IBS), and IBS that was neither diarrhea nor constipation-predominant (5). The latter group was later divided into those with mixed stool patterns (IBS-M) and those with unclassified stool patterns (IBS-U) and those with a predominant stool form were termed IBS-C or IBS-D (7).

It is unclear to what extent the new definitions of IBS overlap with clinically defined IBS. Two studies (8, 9) determined the sensitivity for clinically defined IBS using the Rome III criteria. Engsbro et al. (8) studied 499 patients diagnosed with IBS by general practitioners in Denmark and found that 75% (CI: 71–79%) fulfilled the Rome-III criteria for IBS. Ford et al. (9) studied patients referred for specialist consultation in Canada and 251/365 patients diagnosed with IBS, after exclusion of organic disease using laboratory tests, colonoscopy, and tests for coeliac disease fulfilled the Rome-III criteria and this resulted in a sensitivity for IBS of 69% (CI: 64–73%). Thus, there is evidence to suggest that the use of Rome-III criteria for IBS would exclude from the diagnosis 25–31% of patients with clinically recognized IBS (Figure 1).

## 2. Diseases with IBS-like symptoms

Forty years ago, it was emphasized that IBS was a diagnosis made after exclusion of organic causes for the symptoms. Manning criteria, the Kruis’ model as well as Rome-I and Rome-II criteria all presupposed the absence of a structural or biochemical explanation for the symptoms. However, this requirement was dropped in Rome-III with the motivation that “research will likely confirm that functional gut disorders manifest such [structural or biochemical] findings” (5). From a clinical viewpoint this is not easy to understand, and we retain the view that the term IBS should be reserved for those with symptoms but no clear underlying organic disease.

### 2.1. Lactose malabsorption

Lactose intolerance has been known since long but the mechanisms behind intolerance are still somewhat unclear. The main dietary source of lactose is milk. Lactose is digested to glucose and galactose by the enzyme lactase-phlorizine hydrolase (=lactase) produced by the enterocytes of the small intestine. This enzyme usually disappears at the end of childhood but mutations in the gene *MCM6* (minichromosome maintenance 6), that is involved in the downregulation of lactase production, can lead to persistence of lactase in adult age (10). The most common mutation in Europe and North America is the C/T single nucleotide polymorphism at 13910 base pairs upstream from the gene encoding lactase (11). Worldwide, lactose malabsorption is the rule.

Those that are lactase deficient can develop flatulence, abdominal pain and diarrhea when exposed to dietary lactose. The symptoms overlap with those of IBS and already in the 1970’s it was recommended to test patients with IBS-like symptoms for lactose malabsorption. The oral lactose tolerance test that measured blood glucose levels before and 30 min after drinking a solution of 50 g lactose became the preferred method (12). The test was considered positive if the rise in blood glucose was ≤1.1 mmol/L (<20 mg/dL).

Another test for lactose malabsorption was the lactose hydrogen breath test, which measured the level of hydrogen in expired air before and after ingestion of lactose (13). An increase in breath hydrogen >20 ppm over 3–4 h was taken to indicate that non-digested lactose had reached the colon with bacterial fermentation and hydrogen production.

Today genotyping for LCT-13910 C/T and in those with “wild-type” i.e., CC indicating lactase non-persistence, also the second most common mutation, LCT-22019 G/A would be the most accurate indirect test for lactase activity in Europe (14). However, not everybody with low levels of lactase develops symptoms. Individual factors such as the composition or adaptation of gut microbiota may explain part of the variation (15, 16). Individuals with lactase non-persistence who experience sustained relief from symptoms while on a lactose-free diet would be the relevant subgroup to exclude from being diagnosed with IBS.

No study has yet investigated the effect of a lactose-free diet on patients identified by genotyping. Two studies from the Netherlands (17) and United Kingdom (18) used the oral lactose tolerance test or the lactose hydrogen breath test to define lactose malabsorption in patients with IBS. They found that 24–27% of patients with IBS had lactose malabsorption. In one study 39% of lactose malabsorbers experienced relief of symptoms on a lactose-free diet (18) and in the other study symptoms decreased by 69% (17) and 82% of those with lactose malabsorption remained symptom free on a lactose-free diet 5 years later (19). This means that 10–20% of patients in north-western Europe with IBS may have lactose malabsorption that can be successfully treated with a lactose-free diet.

### 2.2. Other carbohydrate malabsorption

Fructose malabsorption is common, not to say universal since the function of GLUT-5, the apical transporter of fructose into enterocytes is limited. In the presence of glucose, such as after digestion of sucrose to fructose and glucose, another transporter GLUT-2 can be recruited for transport of fructose, but this does not happen when the gut is exposed only to fructose. Fructose malabsorption is perhaps more a problem in areas of the world where fructose has become popular as a sweetener in foods.

Fermentable carbohydrates seem to be of particular importance for symptom generation in patients with IBS and a diet low in FODMAP (fermentable oligo-, di-, and monosaccharides and polyols) has been shown to improve symptoms (20, 21) but so has also more traditional dietary advice, i.e., to encourage a regular meal pattern; avoid large meals; reduce intake of fat; discourage excessive fiber intake, especially insoluble fibers; reduce caffeine; and avoid gas-producing foods, such as beans, cabbage, and onions (22). The response rates using IBS-SSS (IBS Severity Scoring System (23))for studies of FODMAP restriction in patients with IBS has been 50–80% (24).

An interesting finding was that 4% of patients with IBS were found to have rare pathogenic variants in the gene coding for sucrase-isomaltase (25, 26), which implies a reduced ability to digest sucrose and starch. This finding led to a study in which patients with IBS were put on a diet with reduced carbohydrates, starch, and sucrose where 74% of patients responded with a decrease of IBS-SSS with 50 points (27). It was unclear in this as well as previous dietary modification studies to what extent carbohydrate restriction led to complete remission of symptoms.

### 2.3. Celiac disease

Forty years ago, celiac disease (CD) was mainly considered a childhood diagnosis but it was known that CD also occurred in adults, usually with a presentation as idiopathic steatorrhea (28). The diagnosis of celiac disease was based on the presence of villus atrophy in biopsies from small intestinal mucosa obtained by capsule (29). Two developments made it possible to better detect celiac disease. The first was the widespread introduction of gastroscopy in the late 1970’s, which allowed biopsy taking from the duodenum. The other was the discovery of a specific marker for dermatitis herpetiformis and celiac disease in the form of IgA class antibodies to endomysium (30). The two developments made it possible in the mid 1980’s to diagnose celiac disease at an earlier stage, when the clinical presentation overlapped with IBS (31). A meta-analysis of studies comparing the prevalence of celiac disease between patients with IBS and controls showed that pooled prevalence of IgA antibodies to tissue transglutaminase in patients with IBS was 5.7% and the odds ratio for having biopsy proven celiac disease was 4.5 (32).

### 2.4. Microscopic colitis

Since the original report of collagenous colitis by Lindström in 1976 (33) the concept of microscopic colitis broadened to also include patients with lymphocytic colitis (34). The clinical presentation of microscopic colitis is typically chronic, bloodless diarrhea with normal or close to normal findings on colonoscopy (35). The diagnosis can only be made from histopathological examination of biopsies from colon mucosa. Both collagenous colitis and lymphocytic colitis may require biopsies from the right colon. The abnormal collagen band is thickest in the right colon and inflammatory changes are unevenly distributed and may be lacking in the distal bowel (36, 37).

Microscopic colitis has a multifactorial background and several treatments have been tried in microscopic colitis but only budesonide has documented efficacy both for short-term and long-term treatment (38). Although the typical patient with microscopic colitis is a middle-aged or older female with watery diarrhea, the symptoms overlap with those of IBS and 40–50% of patients with microscopic colitis fulfil the Rome-III criteria for IBS (39, 40).

A recent meta-analysis found that the pooled prevalence of microscopic colitis among patients with IBS was 7.4% in cross-sectional studies and 7.1% in case–control studies (41). In the same analysis, the pooled prevalence of microscopic colitis in patients with non-IBS diarrhea was slightly higher (10.9%).

### 2.5. Inflammatory bowel disease

It is well known that patients with ulcerative colitis or Crohn’s disease may have IBS-like symptoms while in remission. A recent, systematic review and meta-analysis found that the pooled prevalence of IBS-like symptoms among patients with inflammatory bowel disease in remission was 32.5% (42). However, few studies have investigated how often inflammatory bowel disease is mistakenly diagnosed as IBS. A large Japanese study of patients undergoing colonoscopy found ulcerative colitis in 3.4% of patients who fulfilled the Rome-III criteria for IBS (43). Neither basic laboratory tests, nor alarm symptoms were considered, hence the study probably overestimated the prevalence of inflammatory bowel disease. Other studies indicate that less than 1.0% of patients with IBS may turn out to have inflammatory bowel disease (44, 45).

### 2.6. Bile acid diarrhea

Bile acids are necessary for water solubilization and digestion of dietary fats. Bile acids are secreted by the liver to the bile, re-absorbed mainly in the ileum and transported to the liver where they are re-secreted into the bile. If reabsorption is impaired due to disease in the ileum or surgical resection then an increased amount of bile acids will enter the colon, where bile acids will stimulate electrolyte and water secretion and give rise to diarrhea. An increased exposure of the colon to bile acids can also occur without disease in the ileum. The first report of such a condition was published in 1973 (46). For several years this was considered a rare manifestation of impaired uptake of bile acids, hence referred to as idiopathic bile acid malabsorption (BAM) (47).

It wasn’t until researchers in the Netherlands could show that the uptake of bile acids was normal or even increased in subjects with BAM (48), that interest changed focus from absorption of bile acids to factors regulating bile acid synthesis (49). In 2009 it was suggested that patients with idiopathic BAM might have an impaired secretion of the protein hormone FGF19 (fibroblast growth factor 19), which is released by the enterocytes for the control of bile acid synthesis in hepatocytes (50). The most common cause of idiopathic BAM now renamed ‘bile acid diarrhea’ is therefore believed to comprise overproduction of bile acids, leading to a larger than normal pool of bile acids that saturates the transport capacity in the distal ileum and leads to increased spill-over of bile acids into the colon.

Measuring the fecal content of bile acids is cumbersome and already in the early 1980’s researchers in Scotland developed an indirect scintigraphy method using a radio-labelled synthetic bile acid ^75^Se-homocholic acid-taurine (SeHCAT) for evaluation of ileal function (51). Newer methods for assessment of bile acid diarrhea include measurements of the serum level of 7α-hydroxy-4-cholesten-3-one (52), sometimes referred to as C4, which reflects the rate of bile acid synthesis (53), and the serum level of FGF19 (54). The SeHCAT-test is not available in the United States, where measurement of fecal bile acids using enzymatic methods (55) or liquid chromatography and mass spectrometry (56) has been used.

Several studies have shown that about 25% of patients with IBS-D have bile acid diarrhea (57, 58). No placebo-controlled studies of the effect of bile acid sequestrants have been done and whether such therapy leads to complete relief from bowel symptoms is not known. For the present review, we have assumed that 80% of those with laboratory findings indicating bile acid diarrhea would respond to treatment, i.e., about 20% of patients with IBS-D or functional diarrhea.

### 2.7. Small intestinal bacterial overgrowth

In 2000 Pimentel and co-workers reported that 78% of patients with IBS might have small intestinal bacterial overgrowth (SIBO) (59). This was concluded from studies using a lactulose hydrogen breath test (LHBT). The authors also reported that 48% of the patients with abnormal LHBT no longer fulfilled the symptom criteria for IBS after treatment with antibiotics (59). This article was met with skepticism not least because the findings of LHBT were not backed by any microbiological data supporting the notion of SIBO. The same research group published a blinded randomized controlled study in which 84% of patients with IBS were reported to have an abnormal LHBT and treatment with neomycin led to a clinical response, defined as at least 50% reduction in a composite score of abdominal pain, diarrhea, and constipation, in 46% of patients with an abnormal LHBT (60). Subsequent research was unable to confirm a difference in LHBT results between patients with IBS and controls (61, 62) and in a study where LHBT was done simultaneously with oro-cecal scintigraphy it was shown that variations in oro-cecal transit time explained the abnormal rise in breath hydrogen measured by LHBT (63).

The role of SIBO in IBS has since remained undefined. A study from our group using massive parallel sequencing to explore the composition of mucosa-associated bacteria in the jejunum found no difference between patients with IBS and healthy controls (64). However, a recent meta-analysis of indirect tests for SIBO found that 31% of patients with IBS had findings indicating SIBO whereas the corresponding figure among controls was 21%. We think that SIBO may have a role in certain patients with IBS-like symptoms and this will be discussed later, but we do not think that SIBO is an important factor for development of IBS.

### 2.8. Exocrine pancreatic insufficiency

Maldigestion due to deficiency of pancreatic enzymes can lead to increased production of gas, abdominal pain, and diarrhea. Exocrine pancreatic insufficiency (EPI) is a differential diagnosis that has been little investigated in relation to IBS. Partly, this is explained by the paucity of readily available tests for exocrine pancreatic function. However, the development of the fecal elastase-1 test has simplified screening for EPI (65).

A study from the United Kingdom investigated 314 patients with IBS-D and found fecal elastase-1 levels <100 μg/g in 19 (6.1%) of these (66). The authors reported a clinical response to enzyme supplementation in 18/19 patients and improvement of abdominal pain in 11/19 patients. However, a study from Australia could not confirm the high prevalence of EPI among patients with IBS and only 2.3% of their patients had fecal elastase-1 levels <100 μg/g (67). It was also pointed out that only a minority of those with low fecal elastase-1 levels had signs of chronic pancreatitis. An obvious source of false low elastase-1 levels is various forms of secretory diarrhea. The contribution of EPI to the differential diagnosis of IBS is probably in the order of 1–2%.

### 2.9. Sodium V1.5 channelopathy

A new pathogenic mechanism for IBS-like symptoms was revealed through a collaborative work between researchers in the US and Europe who found that loss-of-function mutations in *SCN5A* were associated with IBS-like symptoms and that 2.2% of patients with IBS exhibited such mutations (68). *SCN5A* codes for the α-subunit of the voltage-gated Na_V_1.5-channel and such ion channels are present in human smooth muscle cells (69) and the interstitial cells of Cajal (70). Impaired function of the Na_V_1.5-channel may constitute a new target for treatment of IBS-like symptoms.

## 3. Remaining issues

An intriguing feature of IBS is the difference in prevalence between the general population and primary care. Population studies have estimated that 6.2–12.5% of European populations may suffer from symptoms compatible with the diagnosis of IBS (71, 72). However, a detailed analysis of diagnosis registers in primary care found that only 1.2% of patients seen by general practitioners in Sweden received a diagnosis of IBS (73). Other studies have suggested that only 10–30% of people with IBS-like symptoms ever seek medical advice for their symptoms. Those that do seek medical advice are characterized by lower self-reported quality of life and greater levels of anxiety but not significantly different abdominal symptoms (74).

**Figure 2:**
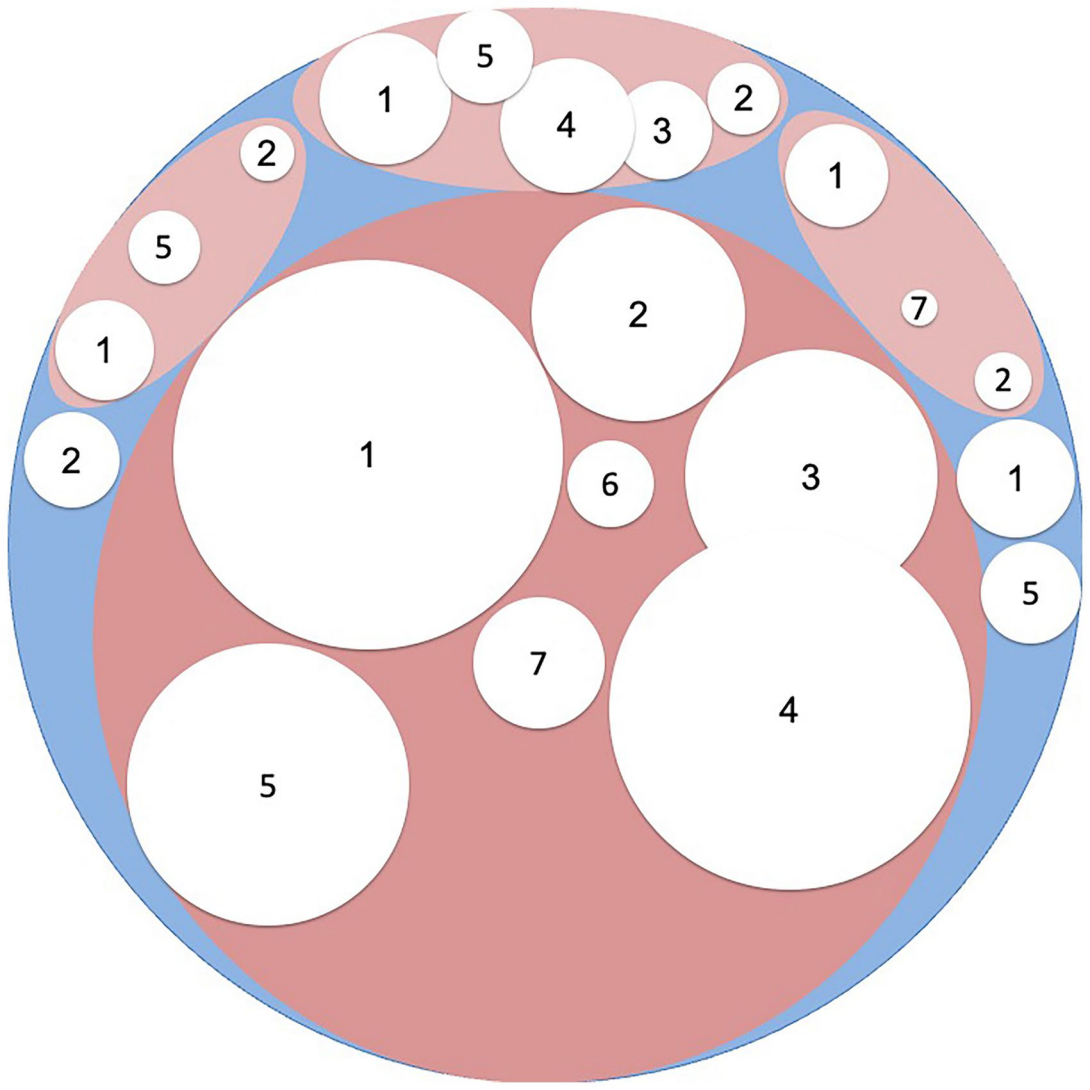
The white areas represent different diagnoses and their relative contribution to the differential diagnosis of irritable bowel syndrome: 1 = Carbohydrate malabsorption (including lactose, fructose and saccharose malabsorption); 2 = Celiac disease; 3 = Microscopic colitis/Inflammatory bowel disease; 4 = Bile acid diarrhea; 5 = Small intestinal bacterial overgrowth; 6 = Exocrine pancreatic insufficiency; 7 = Loss of function mutations in *SCN5A*.

Figure 2 shows the result of advances in the diagnosis of patients with IBS-like symptoms. A little more than a third of patients remain. In our view, it is unlikely that further research will find a single explanation for the remaining patients with IBS. It is much more likely that research will continue to uncover small subgroups with definable causes for IBS-like symptoms.

Genetics have contributed to some of the diagnoses in patients with IBS-like symptoms. In addition to those already mentioned, a genome-wide association study of 53,400 people with IBS and 433,201 controls revealed six genetic susceptibility loci for IBS (75). Four of the six implicated genes (*NCAM1, CADM2, PHF2/FAM120A, DOCK9*) are associated with mood and anxiety disorders and/or expressed in the nervous system. The association was judged to reflect shared etiologic pathways between IBS and anxiety rather than one condition simply causing the other.

Gut microbiota have been extensively investigated in IBS, but research has uncovered subtle changes rather than dramatic alterations in gut microbiota of IBS patients (76). A meta-analysis of case–control-studies of gut microbiota revealed that patients with IBS exhibited increased amounts of *Lactobacillaceae*, *Bacteroides*, and *Enterobacteriaceae* but decreased amounts of *Bifidobacterium* and *Faecalibacterium* (77). It was unclear if observed alterations of microbiota were a product or a cause of IBS and we think that it is unlikely that further analyses of gut microbiota will identify a common culprit for development of IBS.

We propose that two other conditions remain relevant in the differential diagnosis of patients with IBS-like symptoms. This proposition rests upon our clinical experience from a tertiary referral center but we think it is relevant for all patients with IBS-like symptoms who are referred for specialist consultation. The first is connective tissue disorders such as hypermobility spectrum disorders (HSD) and Ehlers-Danlos syndrome (EDS). Patients with HSD or EDS have, in our view, genuine motility disorders that should be treated as such. The second condition is autism spectrum disorders (ASD), which are characterized by disturbances of perception, impairment in social communication and restricted and repetitive interests. A large proportion of patients with ASD report gastrointestinal symptoms (78). Thus, it is unsurprising that a significant proportion of patients with IBS-like symptoms may have ASD.

### 3.1. Connective tissue disorders

Researchers in the United Kingdom highlighted the association between joint hypermobility and functional bowel disorders when they reported that 49% of patients with unexplained gastrointestinal symptoms at a tertiary referral center had clinical evidence of joint hypermobility as assessed by a self-reported 5-point questionnaire (79). A high proportion with joint hypermobility (32%) was also reported from a study of patients with rectal evacuatory dysfunction (80). In a later study the researchers reported joint hypermobility in 33% of patients referred from primary care to GI clinics (81). The authors did not classify their patients according to Rome criteria but in a separate analysis of this material the authors reported that joint hypermobility was present in 35.4% of patients who fulfilled the symptom criteria for IBS (82). Joint hypermobility syndrome is now termed hypermobility spectrum disorders (HSD) (83).

Ehlers-Danlos syndrome (EDS) is an umbrella term for connective tissue disorders with 13 different subtypes (84). The most common subtype is EDS with hypermobility (hEDS). Contrary to the other 12 subtypes, there is no genetic marker for hEDS. The diagnosis of hEDS rests upon a set of criteria, the fulfillment of which creates the dividing line between hEDS and HSD. A retrospective study from the Mayo Clinic in Rochester reported gastrointestinal symptoms in 56% of patients with EDS, and IBS-like symptoms were present in 30.3% of patients with hEDS but also in 18.6% with Classical cEDS and 14.8% with Vascular vEDS (85). Physiological aberrations were common in those that had been investigated: 22.3% had abnormal gastric emptying, 28.3% had abnormal colonic transit, and 60% had a rectal evacuation disorder.

The mechanisms behind IBS-like symptoms in patients with EDS have not been elucidated but EDS is a group of heritable connective tissue disorders caused by mutations affecting collagen formation. Collagen is an important constituent of the gut wall and defects in collagen can be hypothesized to affect gut motility but also intestinal permeability. In our view it is conceptually wrong to label patients IBS if they have EDS or HSD with IBS-like symptoms. We think that the majority of them may have genuinely disordered motility with for example, findings on small bowel manometry qualifying for enteric dysmotility (86). A consequence of such dysmotility is an increased risk for bacterial overgrowth in the small bowel. Patients with EDS or HSD may constitute the main group with IBS-like symptoms who are at risk for small intestinal bacterial overgrowth.

A case–control study of hospitalized patients in the United States investigated the prevalence of gastrointestinal, cardiovascular, autonomic, and allergic manifestations in Ehlers-Danlos syndrome (87). Gastrointestinal conditions were found in 44% of EDS patients and 14% of controls. IBS (odds ratio = 7.44) and gastroparesis (odds ratio = 12.26) were strongly associated with EDS. The study also found a significant association of food allergy (odds ratio = 3.88) and autonomic dysfunction (odds ratio = 4.45) with EDS. In particular, the postural tachycardia syndrome (PoTS), neurocardiogenic syncope, and orthostatic hypotension are common in patients with EDS (88). In a large case–control study using an online survey of persons with HSD/hEDS, 98% met the criteria for functional gastrointestinal disorders according to Rome-IV (89). The criteria for functional bowel disorders were fulfilled by 90% of patients with HSD/hEDS compared to 40% of population controls.

Little is yet known about therapeutic alternatives in EDS-associated IBS-like symptoms but it is reasonable to assume that this subgroup of patients with IBS-like symptoms may differ from other subgroups of IBS regarding therapeutic responses. It is possible, yet not shown that the use of antibiotics and prokinetics may have a more important place in therapy. We have used bedtime subcutaneous injections of octreotide, 25–50 μg after an initial course of antibiotics, to prevent further small intestinal bacterial overgrowth with some success in patients with HSD/hEDS and enteric dysmotility (86). Food hypersensitivity is also frequent in this group of patients. Antihistamines as well as mast cell stabilizers may have a role in the treatment of such patients but firm data from randomized clinical trials are lacking.

### 3.2. Autism spectrum disorders

Autism spectrum disorders (ASD) is a neurodevelopmental disorder characterized by deficits in social communication and social interaction, in addition to restricted, repetitive patterns of behavior, interests, or activities (90). Many are diagnosed early in life, but high functioning ASD can remain undetected throughout childhood and adolescence. There is a plethora of studies dealing with gastrointestinal symptoms among children with ASD (91, 92). However, there is much less in the literature on the association of ASD with gastrointestinal symptoms in adults. One study of 107 adults with ASD found that 86% had at least one gastrointestinal symptom but 54% had at least three symptoms and diarrhea was reported by 62% of participants (93). Another study utilized Wisconsin Medicaid claims data to study the co-occurrence of physical and mental health conditions in 143 adults with ASD aged 40–88 years (94). The study reported that 50% had been diagnosed with gastrointestinal disorders. No difference was found between patients with or without intellectual disability.

We propose that ASD co-morbidity is a significant contributor to IBS as well as other functional gastrointestinal disorders, at least in tertiary care. Those with a known ASD since childhood is seldom a problem but so are undiagnosed patients with high functioning ASD. Such patients have often been extensively investigated and they have usually seen many health care providers. They cannot accept the diagnosis and they often claim that they have something unusual that has been missed. Today the Internet provides the patients with extensive opportunities to dispute their doctor’s advice. Another feature of ASD is perception disturbances with atypical sensory processing reported by 82 to 97% of children with autism (95). An increased conscious sensitivity to signals from the gastrointestinal tract has long been a hallmark of patients with functional gastrointestinal disorders but it can also be viewed as disturbed perception.

Our clinical experience from a tertiary referral center is that a significant proportion of our “difficult” patients with IBS or IBS-like symptoms may have ASD rather than a gastrointestinal disorder. When we looked at new referrals during 2022, we found that 12% of those who had symptoms without an organic correlate (irrespective of whether symptoms coincided with symptom criteria for IBS) had already been diagnosed with ASD as children. Our clinical suspicion was that unrecorded ASD might be even more prevalent. We also think that ASD may explain why many doctors fear the meeting with a patient that does not accept the diagnosis of a functional gastrointestinal disorder. It is difficult, not to say impossible, to gain acceptance for explanations of symptoms. Often, the patient has some other theory and asks for further investigations to rule out any of a set of far-fetched diagnoses or has an explanation for symptoms that does not fit with any known disease.

Self-imposed dietary restrictions are also common in this group of patients, not seldom with a potential for malnutrition. If a patient claims that he can eat only cooked broccoli or cauliflower, butter and Himalaya salt, the odds favor ASD instead of multiple food allergy.

## 4. Conclusion

Most patients with IBS-like symptoms have been shown to suffer from treatable conditions explaining their symptoms. Future research may continue to uncover small subgroups with treatable causes for IBS-like symptoms. However, we think that research should focus on two areas, yet little examined, in relation to IBS and IBS-like symptoms. The first area concerns the role of connective tissue disorders in the generation of IBS-like symptoms. Studies are needed to elucidate the mechanisms by which connective tissue disorders lead to gastrointestinal dysfunction and symptom generation. Gastrointestinal motility, autonomic dysfunction, intestinal permeability, immune activation, and intestinal microbiota are examples of research areas in which current knowledge is insufficient to understand the role of such disorders.

The second area is to understand the role of ASD in care seeking behavior as well as presentation of IBS-like symptoms. We think it is important to find out why some people with IBS-like symptoms become patients whereas others do not. Clearly if you think that “Physical symptoms are not normal and always a sign of serious disease,” you will be more likely to seek medical advice for your symptoms than if you think otherwise (96). There is a dilemma for doctors and other health care providers in the choice of strategy for a patient with IBS-like symptoms. We think it is highly relevant to include high functioning ASD in the differential diagnosis of a patient with unclear symptoms. However, early recognition of ASD is a challenge not least because we are taught to develop a good doctor-patient relationship involving mutual participation and a focus on patient centered medicine. We think that we need to improve our skills to communicate with patients who have high functioning ASD and IBS-like symptoms. We doubt that labelling such patients “IBS” is the best strategy. We think it would be better to use the correct diagnostic label.

## Data availability statement

The original contributions presented in the study are included in the article/supplementary material, further inquiries can be directed to the corresponding author.

## Author contributions

All authors listed have made a substantial, direct, and intellectual contribution to the work and approved it for publication.

## Conflict of interest

The authors declare that the research was conducted in the absence of any commercial or financial relationships that could be construed as a potential conflict of interest.

## Publisher’s note

All claims expressed in this article are solely those of the authors and do not necessarily represent those of their affiliated organizations, or those of the publisher, the editors and the reviewers. Any product that may be evaluated in this article, or claim that may be made by its manufacturer, is not guaranteed or endorsed by the publisher.
